# Classification and Quantitative Characterization of Archean Metamorphic Buried Hill Reservoirs in the Bohai Sea

**DOI:** 10.1021/acsomega.3c04890

**Published:** 2023-09-13

**Authors:** Shuyue Ren, Dingyou Lv, Jian Yi, Xuanlong Shan, Xiaojian Liu, Bohan Zhu, Wei Wang, Pengcheng Liu

**Affiliations:** † College of Earth Science, Jilin University, Changchun 130061, China; ‡ Tianjin Branch of China National Offshore Oil Corporation Ltd., Tianjin 300450, China

## Abstract

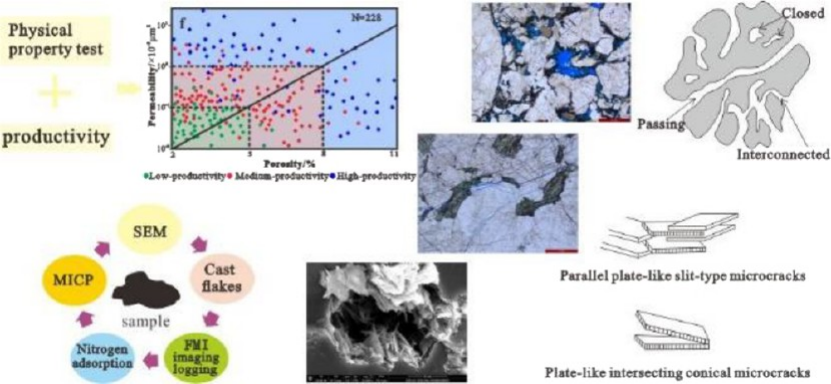

Based on productivity test data and physical property test results from multiple wells, a classification scheme of Archean metamorphic buried hill reservoirs in the Bohai Sea is established by means of mathematical function fitting. By combining data from cores, casting thin sections, scanning electron microscopy, imaging logging, and high-pressure mercury injection and nitrogen adsorption tests, we clarified the reservoir composition and pore structure characteristics of different types of reservoirs are clarified. Furthermore, taking the BZ19-6 and 13-2 wells in the Archean metamorphic buried hills as an example, the development sites of different types of reservoirs are analyzed and the reservoir development model is established. The results show that the Archean metamorphic buried hill reservoirs in the Bohai Sea can be divided into three categories and six subcategories, including type I reservoirs with porosities greater than 8% or permeabilities greater than 1 × 10^–3^ μm^2^ and type II reservoirs with porosities of 5–8% or permeabilities in the range of 0.1–1 × 10^–3^ μm^2^. Reservoirs with porosities of 2–5% and permeabilities of 0.01–0.1 × 10^–3^ μm^2^ are type III reservoirs. Each type of reservoir can be further divided into a fracture-pore type and a fracture type according to the relative contribution of the porosity and permeability to the reservoir. From type I to type III, the dissolution degree and fracture development gradually weaken, the pore size gradually decreases, and the pore volume gradually decreases. The distribution of favorable reservoirs is comprehensively controlled by weathering and tectonic transformation. The presence of a weathered glutenite zone, weathered leaching zone, or weathered disintegration zone is favorable for the development of type I reservoirs in the weathering crust. In the inner part of the buried hill, the presence of a fracture zone with a thickness of more than 10 m or a dense fracture zone with a thickness of more than 40 m is favorable for the formation of type I reservoirs.

## 1 Introduction

With the continuous expansion of oil and gas exploration to the deep and ultradeep layers, the Archean metamorphic buried hill in the Bohai Sea has attracted much attention in recent years as an important field of oil and gas production in the Bohai Bay Basin.^1−4^ Archaean buried hills are widely distributed in the Bohai Sea. The discovery of giant oil-gas fields such as JZ 25-1 South, PL 9-1, BZ19-6, BZ13-2, and others has shown the great exploration potential of buried metamorphic rock hills in the Bohai Bay Basin.^5,6^ The metamorphic buried hill reservoir has no primary pores, the reservoir space is mainly secondary pores and fractures formed by later reconstruction, and the development of the reservoir is not affected by the burial depth. The oil and gas reservoirs are characterized by differential enrichment in vertical and horizontal directions. The strong heterogeneity of the reservoir leads to great differences in the productivity tests of different exploration wells. The classification and evaluation of metamorphic rock reservoirs and the clarified relationship between different types of reservoirs and productivity can significantly improve the exploration efficiency.

Research on metamorphic rock reservoirs has been reported upon by scholars at home and abroad. The reports mainly focus on the types of metamorphic rock reservoir space, control factors, and vertical zoning characteristics. Previous studies have shown that the reservoir space of metamorphic buried hills is dominated by structural fractures and dissolved pores.^7−10^ Reservoirs are divided into three types according to the combination of the reservoir space: pore type, fracture type, and fracture-pore type.^9,10^ Weathering and tectonic movement are the main factors that transform tight zone metamorphic rock into reservoir space.^10^ The strength of weathering controls the thickness of the weathered crust reservoirs, and tectonic movement controls the development of the fractures in reservoirs.^9,11^ At the same time, the difference in weathering and tectonic activity causes metamorphic buried hill reservoirs to have zoning characteristics in the vertical direction.^10−13^ However, the production of more than 40 wells drilled in the Archean area of the Bohai Sea has found that pore, fracture, and fracture-pore reservoirs can become high-quality reservoirs, and there are also low-productivity or even ineffective reservoirs. In other words, the classification scheme cannot be linked with the productivity of metamorphic rock reservoirs and cannot truly distinguish the advantages and disadvantages of reservoirs or effectively classify and evaluate reservoirs. In other words, the classification scheme cannot accurately determine the vertical development site and spatial distribution pattern of reservoirs and cannot accurately guide the development of favorable reservoirs in the future.

In view of this, this study focuses on typical drilling of the Archaean buried hills in the Bohai Sea based on the single-well productivity, uses the Gaussian curve to fit the porosity and permeability curve, and uses the mathematical method to analyze the contribution of different pore combination conditions to the single-well productivity. The porosity and permeability boundaries of high- (daily gas production >10 × 10^4^ m^3^/d), moderate- (5–10 × 10^4^ m^3^/d), low- (<5 × 10^4^ m^3^/d), and no-productivity reservoirs are determined, and reservoir classification and evaluation standards are established. The reservoir space and pore structure characteristics of each type of reservoir are quantitatively characterized by cores, sidewall cores, casting thin sections, imaging logging, high-pressure mercury injection, and nitrogen adsorption experiments, and the development sites of different types of reservoirs are further analyzed.

## 2 Geological Setting

**Figure 1:**
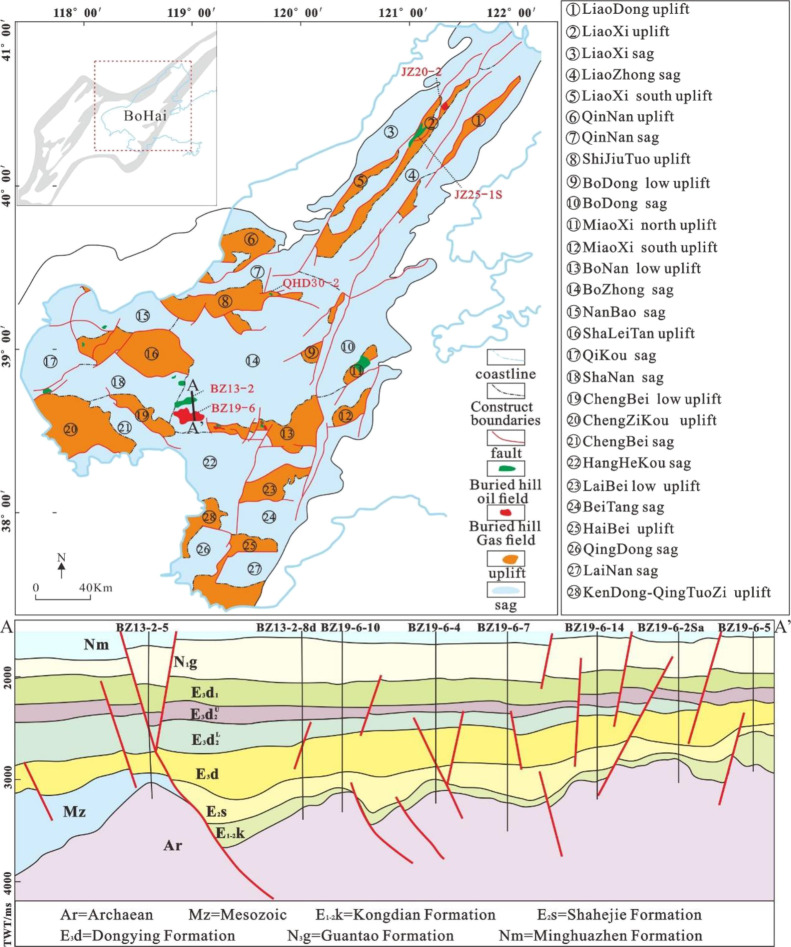
Location, structural pattern, and distribution of buried hill oil and gas fields in the Bohai Sea (modified by a previous study^17^).

**Figure 2:**
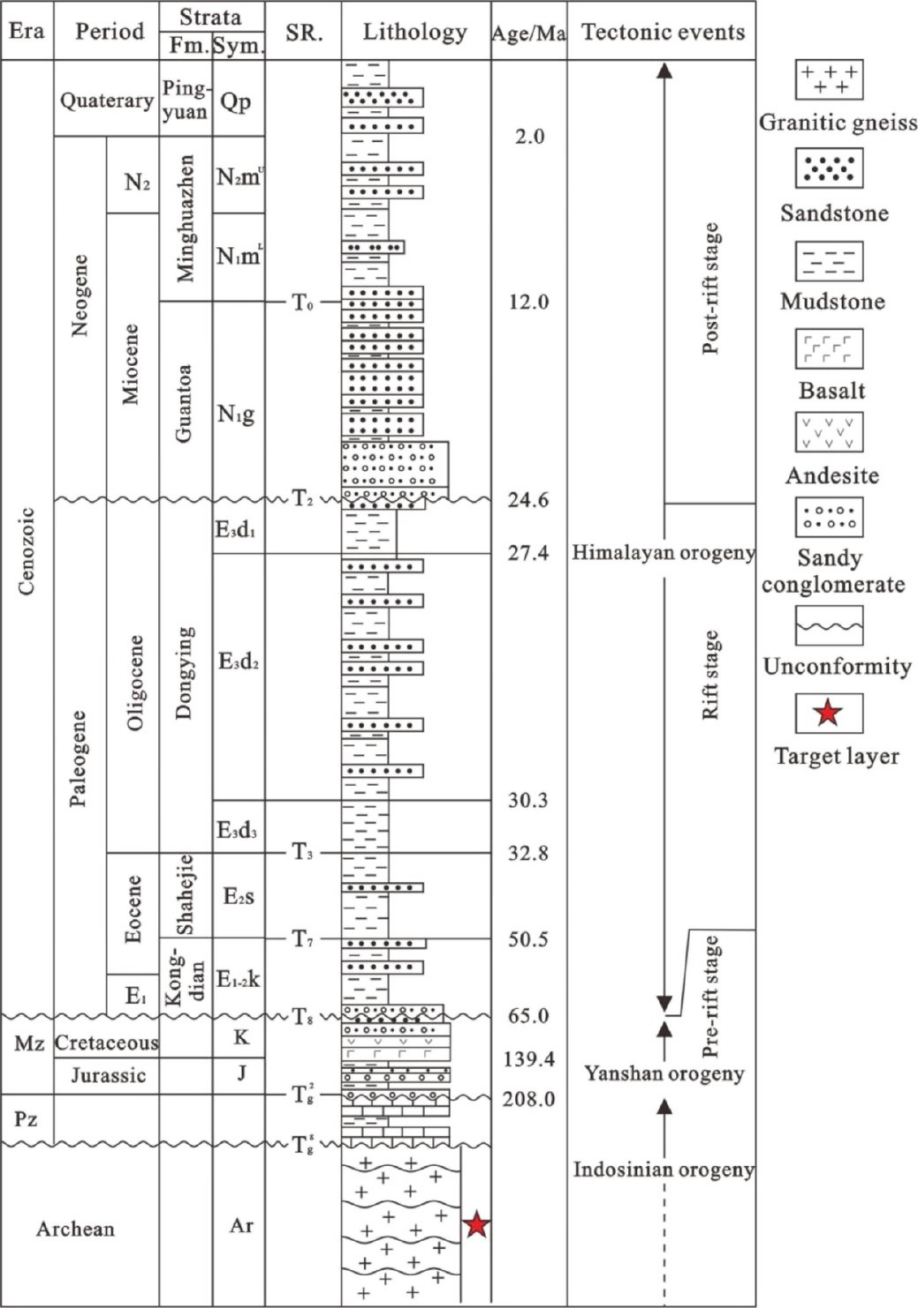
Stratigraphic column map of the Bohai Sea.

The Bohai Bay Basin is an important oil and gas basin in eastern China, with an area of approximately 20 × 10^4^ km^2^, and the annual crude oil production is more than 7000 × 10^4^ t, which is more than 1/3 of the total crude oil production in China.^1,2,5,7^ The basin is a multistage fault depression basin formed by tectonic movements such as the Indosinian, Yanshanian, and Himalayan periods, with structural characteristics of alternating uplift and depression.^14^ The Bohai Sea is in the eastern part of the Bohai Bay Basin. It is surrounded by the Jiaoliao Uplift to the east, the Jiyang Depression to the south, the Huanghua Depression to the west, and the Xialiaohe Depression to the north.^5,15^ The pre-Paleogene era strata in the Bohai Sea include Archean, Proterozoic, Paleozoic, Mesozoic, and Cenozoic strata from the bottom to top. The basement is Archaean, and the lithology of the basement is mainly metamorphic rock, including mixed granite, mixed gneiss, and gneiss^16^ (Figures 1 and 2).

The formation of buried hills in the Bohai Sea mainly includes four stages.^15,18^ ① At the sedimentary construction stage during the pre-Indosinian, the tectonic movement was dominated by overall vertical uplift, and the structural style was not developed. ② In the tectonic development stage of buried hills during the Indosinian and early Yanshanian, the nearly north–south compressive stress widely developed structural styles such as folds and thrusts in the study area. ③ The formation stage of the initial Yanshanian pattern in the middle to late period, during strong magmatic activity and extension, combined with left-lateral strike slip, modified the early folds and thrust structures to form a fault-block buried hill pattern. ④ The stage from Cenozoic to reconstruction burial includes Palaeogene inherited rifting and Neogene thermal subsidence burial. Episodic extension occurred in the Palaeogene and generally inherited the tectonic framework of the Late Jurassic-Early Cretaceous intracontinental rifting period. The early sag-controlling faults were extended and superimposed, block faulting and tilting caused the buried hill to be further uplifted, and the structural scale was expanded. At the beginning of the Miocene, the Bohai Sea entered the stage of fault-depression transformation; rifting was terminated, the postrift thermal subsidence was transformed, and the buried hill entered the stage of stable burial (Figure 2).

Metamorphic buried hills are distributed in various uplifts and depressions in the basin. Although metamorphic buried hills have experienced the same tectonic evolution stage, the manifestations of tectonic movements in each stage are different in different regions, which makes the types of buried hills in the basin different. The reservoir characteristics and development patterns vary significantly in different types of buried hills. The experimental data of this study cover the tectonic area, which mainly includes the JZ25-1S structural area in the middle section of the Liaoxi Uplift, the JZ20-2 structural area in the north section of the Liaoxi Uplift, the BZ19-6 structural area in the southwest of the Bozhong Depression, the BZ13-2 structural area, and the QHD30-2 structural area at the west high point of the Shijiutuo Uplift (Figure 1).

## 3 Experimental Data and Methods

### 3.1 Samples

The experimental samples in this study are metamorphic rock samples from 32 wells located in Archean metamorphic buried hills in the Bohai Sea. The samples cover five tectonic areas, namely, BZ19-6, BZ13-2, JZ25-1S, JZ25-3, and QHD. Some samples are buried at depths of more than 4000 m, and some samples are buried at depths of 1500–2500 m. These samples include buried hills exposed in the Archaeozoic, as well as buried hills covered by the Paleozoic or Mesozoic. The experimental test results can represent the reservoir characteristics of the buried Archaeozoic metamorphic hills in the Bohai Sea.

### 3.2 Observation of Cores, Sidewall Cores, and Casting Sections

To identify and count the large-scale pores and fractures, more than 180 m of cores were observed in the core database, taken from 20 wells. To observe and identify the microscopic spatial characteristics of the reservoir, 709 cast sections were collected. For this study, imaging logging data of tens of thousands of meters from 21 wells were collected with the aim of identifying and statistically analyzing small-scale fractures. The samples and data above are provided by the Tianjin Branch of China National Offshore Oil Corporation. Cast flakes are made by injecting blue resin into the pores of rocks in a vacuum state or under pressure and grinding them after the liquid glue solidifies. The blue resin dye can better identify the pores and fracture distribution development characteristics of rocks. Fifty sample points were selected from the observed casting flakes to observe the very small pores and cracks that cannot be detected at the scale of the casting flakes by scanning electron microscopy. The observation of casting thin sections was completed by a ZEISS polarizing microscope, and the observation of SEM was completed by a Quanta 200 F SEM system.

### 3.3 Pore Permeability Test

Cores with high-, moderate-, and low-productivity capacities were selected from the observed cores for sampling. The sampling specifications were small cylinders with diameters of 2.5 cm and lengths of 3 cm. The routine core analysis and testing on the plungers were completed by using the QKY-2 gas porosity measuring instrument to test the porosity using nitrogen as the displacement medium and the STY-2 gas permeability measuring instrument to test the permeability using nitrogen as the medium. These experiments were completed in the Key Laboratory of Xi’an Petroleum University.

### 3.4 High-Pressure Mercury Intrusion

The high-pressure mercury intrusion experiment incorporated a Conta PoreMaster33 mercury injection tester, with a maximum pressure input of 33,000 psi and a pore distribution measurement range of 1080–0.005 μm. The parameter analysis was based on GB/T29171-2012. The radius of the pore throat was obtained through Washburn’s equation. These experiments were completed in the Key Laboratory of Xi’an Petroleum University.

### 3.5 Low-Temperature Nitrogen Adsorption

The low-temperature nitrogen adsorption desorption test was conducted using the TriStar II3020 fully automatic specific surface area and pore analyzer produced by McMuritick Instruments in the United States. The instrument has a pore size measurement range of 0.35–500 nm and is analyzed based on the “Static adsorption capacity method for determining the specific surface area and pore size distribution of rocks” (SY/T6154-2019) standard. The pore volume was obtained by using the Barrett–Joyner–Halenda (BJH) theoretical model. These experiments were completed in the Key Laboratory of Xi’an Petroleum University.

## 4 Results

The oil and gas reservoir evaluation method (SY/T6285-2011) defines the classification criteria for the porosity and permeability of metamorphic rock reservoirs, but it is not applicable to the evaluation of unconventional low-porosity and low-permeability reservoirs with complex pore structures present in the study area. In the classification and evaluation of unconventional reservoirs, the boundaries of physical parameters such as the pores and permeability of different types of reservoirs are usually determined based on the capacity of the reservoirs, and then, the evaluation criteria for reservoir classification are established to classify different types of reservoirs. Therefore, establishing the relationship between reservoir physical properties and drilling productivity is the basis for performing reservoir classification and evaluation.

### 4.1 Lower Limit of Physical Properties

**Figure 3:**
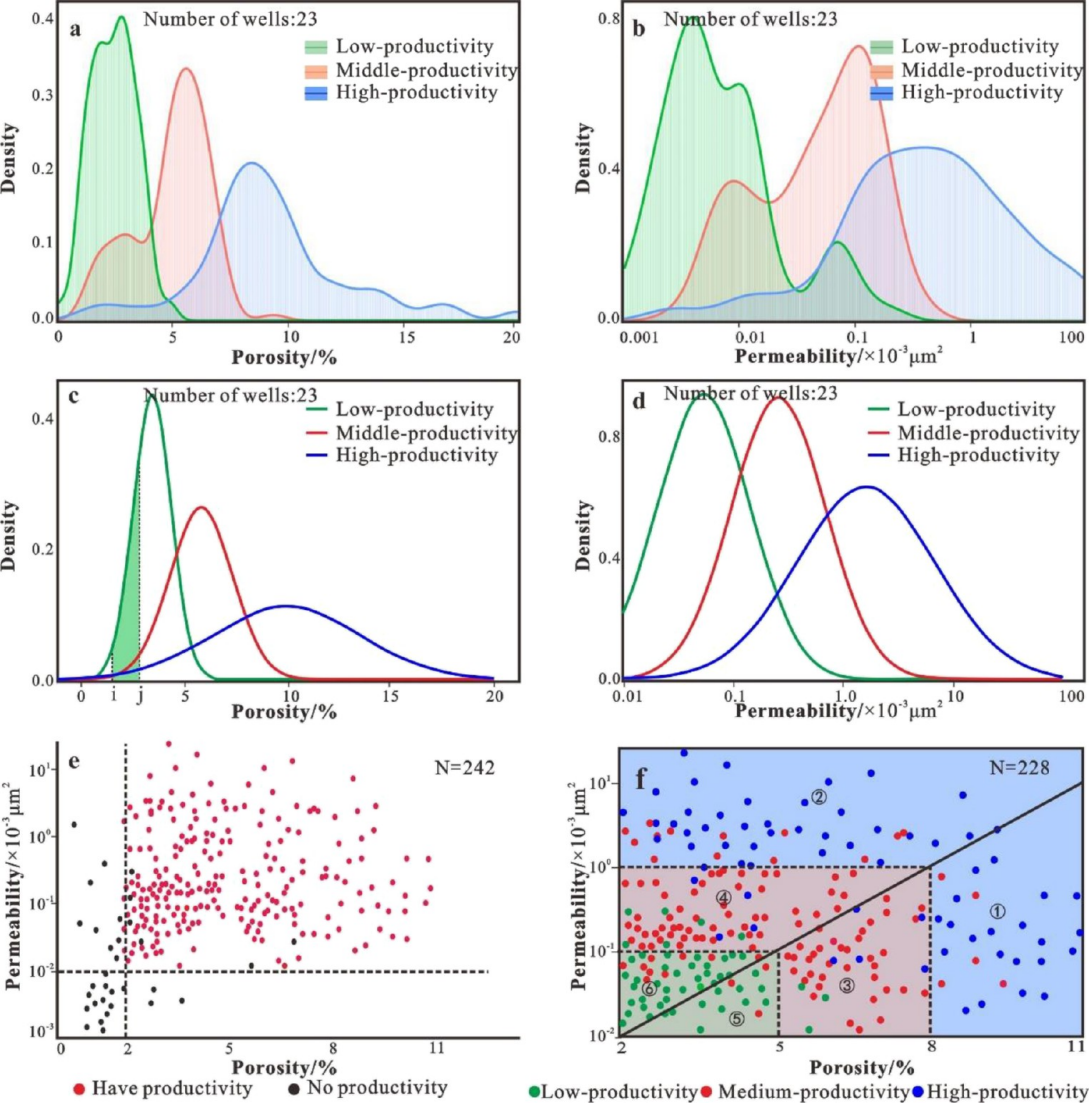
Reservoir classification map of the Archean metamorphic buried hill in the Bohai Sea. (a) Distribution of the porosity density curves of high-, medium-, and low-productivity section samples. (b) Distribution of the permeability density curves of high-, medium-, and low-productivity section samples. (c) Gaussian function fitting of the porosity density curve. (d) Gaussian function fitting of the permeability density curve. (e) Logging interpretation data to determine the lower limit of the physical properties. (f) Reservoir classification criteria (① indicates a type I fracture-pore reservoir. ② indicates a type I fractured reservoir. ③ indicates a type II fracture-pore reservoir. ④ indicates a type II fractured reservoir. ⑤ indicates a type III fracture-pore reservoir. ⑥ indicates a type III fractured reservoir).

To classify and evaluate reservoirs, the first step is to determine the lower limit of the physical properties, which is the boundary between effective reservoirs and noneffective reservoirs. Usually, the lower limit of the physical properties can be determined by using the corresponding relationship between the logging interpretation data and the porosity and permeability properties of oil and gas reservoirs.^19−21^ The physical property testing data of metamorphic buried hills in the research area will be divided into two types according to the measurement solution: oil and gas layers and nonoil and gas layers (Figure 3e). Statistics show that the porosity of metamorphic rocks in the nonoil and gas layers is less than 2%, and the permeability is less than 0.01 × 10^–3^ μm^2^. More specifically, metamorphic rocks with porosities less than 2% and permeability values smaller than 0.01 × 10^–3^ μm^2^ cannot be used as effective reservoirs. Therefore, the lower limit was determined to be porosity less than 2% and permeability less than 0.01 × 10^–3^ μm^2^ for the physical properties of the metamorphic buried hill reservoir in the study area.

### 4.2 Effective Reservoir Classification

The reservoir types are further classified according to the corresponding relationship between porosity and permeability data and productivity for effective reservoirs with porosities greater than 2% and permeabilities greater than 0.01 × 10^–3^μm^2^. Obtaining this connection is very simple for drilling wells that are stratified by capacity segments. However, the Archean metamorphic buried hill reservoir in the Bohai Bay Basin is an unconventional reservoir with extremely developed fractures. To expand the gas gathering area, open hole completion and comprehensive testing of the whole well section are adopted, and it is therefore impossible to directly obtain the one-to-one correspondence between different production sections and the physical properties of the reservoir, which has caused great difficulties in the establishment of the classification and evaluation criteria for Archean metamorphic buried hill reservoirs in this area. To overcome this difficulty, the drilling in the research area was divided into three categories: high productivity (daily gas production >10 × 10^4^ m^3^/d), moderate productivity (5–10 × 10^4^ m^3^/d), and low productivity (<5 × 10^4^ m^3^/d) (in particular, there is no clear and consistent classification scheme for the productivity of different reservoirs in different basins worldwide; the productivity classification of this study is based on the actual production of Archean metamorphic reservoirs in the CNOOC Tianjin Branch). The distribution characteristics of the porosity and permeability in different production capacity drilling were analyzed (Figure 3a,b), and the reservoir types were classified based on the distribution characteristics of the porosity and permeability in different production capacity drilling. The analysis shows that the porosity and permeability distribution range in low-, moderate-, and high-productivity wells is wide, with the difference being that the peak appears at different positions but the curves have overlapping parts. Therefore, the mechanism for determining the pore permeability distribution characteristics of low-, medium-, and high-productivity drilling needs to be comprehensively utilized to determine the physical property boundaries of different types of reservoirs.

To effectively reservoir the metamorphic rocks in the study area (with porosity greater than 2% and permeability greater than 0.01 × 10^–3^ μm^2^), they are further divided into three types of reservoirs, I–III, as the target. First, Gaussian function fitting is performed on the distribution curves of pore permeability density corresponding to three types of drilling wells with different production capacities of low, medium, and high.
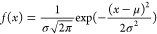
1
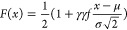
2where *f*(*x*) is the Gaussian function (frequency density curve), *F*(*x*) is the original function of the Gaussian function (frequency cumulative distribution curve), σ is the variance of the data, μ is the mean, and *x* is the data set of porosity and permeability for low-, medium-, and high-productivity reservoirs. The Gaussian distribution curves of porosity *f*φ_1_(*x*), *f*φ_2_(*x*), and *f*φ_3_(*x*) (Figure 3c) and the permeability Gaussian curves *f*_k1_(*x*), *f*_k2_(*x*), and *f*_k3_(*x*) were fitted for the three types of productivity drilling (Figure 3d). Taking two points on the *X*-axis, *x* = *x_i_*, *x* = *x_j_*, respectively, then *x* = *x_i_*, *x* = *x_j_*, and the area enclosed by the *X*-axis, the Gaussian curve is denoted as ∫_*i*_^*j*^*f*(*x*) which represents the proportion of the porosity or the permeability between *x_i_* and *x_j_* (Figure 3c), that is, *F*(*x_j_*)–*F*(*x_i_*).

Then, the function analysis method is used to determine the classification boundary of type I–III reservoirs.

Taking the determination of the porosity boundary between type II and type III reservoirs as an example, we describe the methods for determining the physical boundaries of different types of reservoirs. To determine the porosity boundary between type II and type III reservoirs, we need to find an equilibrium point *x*_1_ on the *X*-axis so that the low-productivity Gaussian curve *f*φ_1_(*x*) falls to the left of *x* = *x*_1_ as much as possible; that is, the low-productivity values are located within the range of type III reservoirs as much as possible. At the same time, the middle Gaussian curve *f*φ_2_(*x*) and the high Gaussian curve *f*φ_3_(*x*) values fall to the right of *x* = *x*_1_ as much as possible; that is, the moderate and high productivity values fall within the range of type III reservoirs as little as possible. At this point, *x*_1_ is the boundary value of the porosity for type II and type III reservoirs. *S*_1_ represents the area of *f*φ_1_(*x*) at 0–*x*_1_, *S*_2_ represents the area of *f*φ_2_(*x*) at *x*_1_–∞, and *S*_3_ represents the area of *f*φ_3_(*x*) at *x*_1_–∞. When *x* = *x*_1_, the function *G*(*x*)= *S*_1_+ *S*_2+_*S*_3_ (formula 3) should obtain the maximum value.
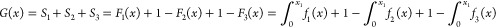
3

Derivation of *G*(*x*) is
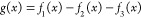


Let *G*′(*x*)=0, that is
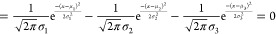


where μ_1_, μ_2_, and μ_3_ are the average porosity or permeability of the low-, medium-, and high-yield reservoirs, respectively, and σ_1_, σ_2_, and σ_3_ are the standard deviations of the porosity or permeability of the low-, medium-, and high-yield reservoirs, respectively.

The solution of this function is based on the idea of dichotomy root-seeking and is programmed in Python. The idea of dichotomy is to continuously narrow the interval for finding roots in the process of dichotomy; that is, if the equation *H*(*x*)=0 has roots in the interval [*a*, *b*], then the signs of *H*(*a*) and *H*(*b*) must be opposite, and then, the midpoint of a and b is taken and then divide the interval for finding roots into two halves, judge which interval the root is in, and then continuously repeat the dichotomy process to keep narrowing the interval containing roots until the root is found or it is determined to be close enough to the root. The dichotomy root calculation result: *X*φ_1_ is 4.52345 and rounded to *X*φ_1_ ≈ 5.

Using this method, we sequentially obtain that the boundary *X*φ_2_ between type I and type II reservoirs is 7.96679, rounded to *X*φ_2_ ≈ 8. The boundary value *X*_K1_ between type II and type III permeabilities is 0.10715, rounded to *X*_K1_ ≈ 0.1. The boundary value of permeability *X*_K2_ between type I and II reservoirs is 0.74148, rounded to *X*_K2_ ≈ 1.

**Figure 4:**
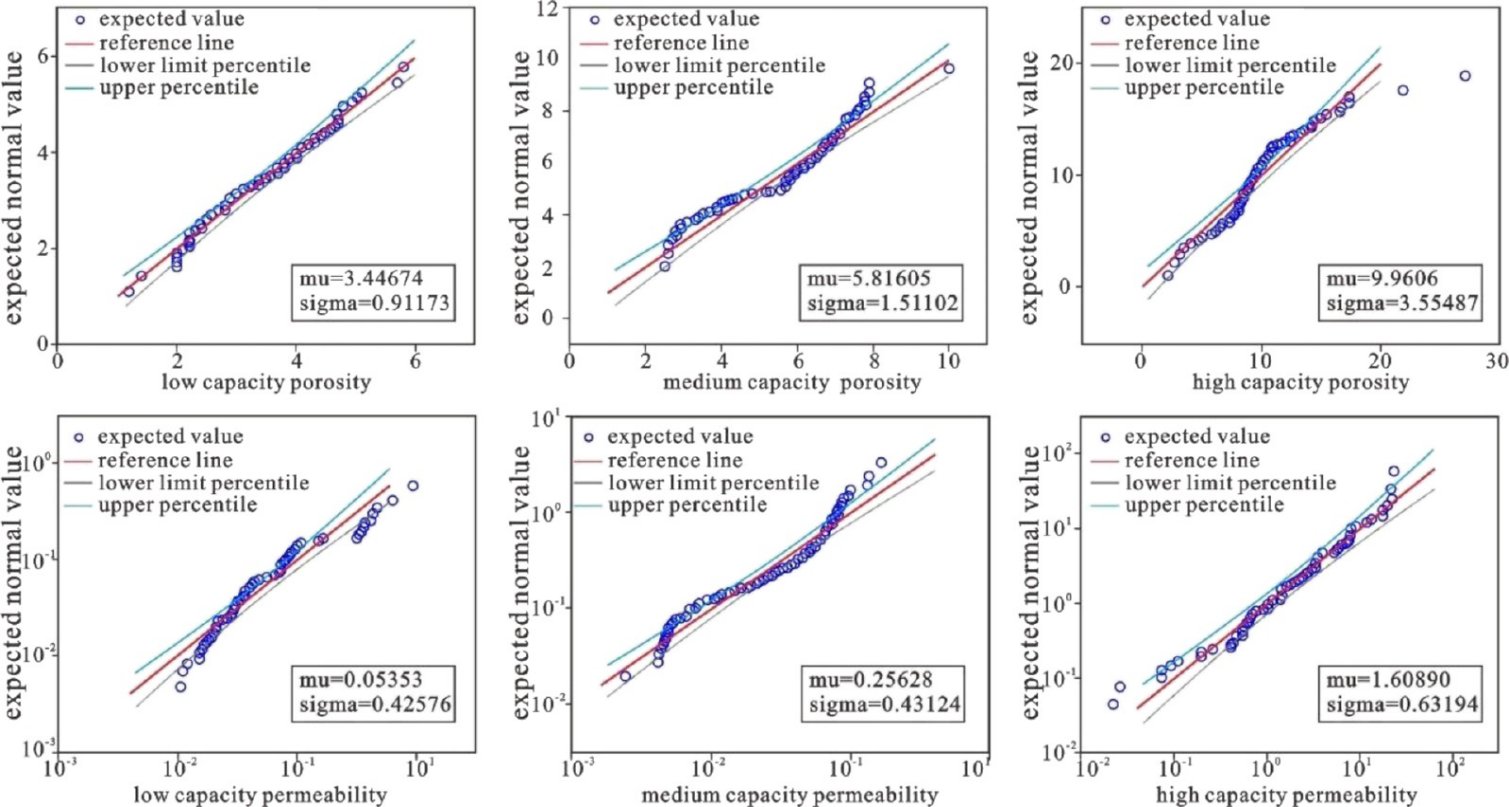
QQPLOTs for the porosity and permeability data of low-, medium-, and high-productivity wells.

To determine the rationality of the Gaussian function fitting, a distribution test plot (QQPLOT) is used (Figure 4). The principle of QQPLOT plot testing is to compare the quantiles of the test sample data with known distributions. When the reference line is close to the straight line *Y* = *X*, it indicates that the distribution of the original sample values is highly similar to the distribution of the fitted curve and the reliability of the fitting curve is higher.

According to the porosity and permeability boundaries of the type I–III reservoirs in the Archaean buried hills in the study area determined above, we can determine the distribution intervals of the type I–III reservoirs and the nonreservoirs on the porosity and permeability scatter plot. It can be seen from the pore permeability point diagram that the metamorphic rock reservoir is a fracture-pore dual-media reservoir, and its porosity and permeability are poorly correlated. Type I and II reservoirs are mainly distributed in the orthogonal ruler interval. For type I reservoirs, when the permeability is greater than 1 × 10^–3^ μm^2^, it can still become type I, even if the porosity is <8%. When the porosity is >8%, even if the permeability <1 × 10^–3^ μm^2^, the reservoir can still become a type I reservoir. Thus, type I reservoirs only need to meet the requirements of porosity >8% or permeability >1 × 10^–3^ μm^2^. Similarly, type II reservoirs only need to meet one of the requirements of porosity between 5 and 8% or permeability between 0.1 and 1 × 10^–3^ μm^2^. Type III reservoirs are distributed in a rectangular range composed of 2–5% porosity and 0.01–0.1 × 10^–3^ μm^2^ permeability intervals. Based on whether different types of reservoirs are dominated by pores or fractures, we further classify the type I–III reservoirs into two types, the fracture-pore type and the fracture type (Figure 3f, Table 1).

**Table 1:** Classification and Evaluation of Archean Metamorphic Buried Hill Reservoirs in the Bohai Sea

type	subtype	Pphysical property interval	reservoir space	productivity
I	fracture-pore	porosity >8%	intergranular pores and dissolution pores are dominant.	high
	fractured	permeability >1 × 10^–3^ μm^2^	large-scale structural fractures and dissolution expansion fractures	high
II	fracture-pore	porosity is between 5 and 8%	dissolution pores are mainly	medium
	fractured	permeability is between 0.1 and 1 × 10^–3^ μm^2^	small-scale structural fractures and semi filled fractures	medium
III	fracture-pore	porosity is between 2 and 5%	micropore based	low
	fractured	permeability is between 0.01 and 0.1 × 10^–3^ μm^2^	the microfractures are dominant	low

## 5 Discussion

### 5.1 Reservoir Space Characteristics of Different Types of Reservoirs

The metamorphic rock reservoir has no primary pores, and the reservoir space is mainly composed of secondary pores and fractures.

**Figure 5:**
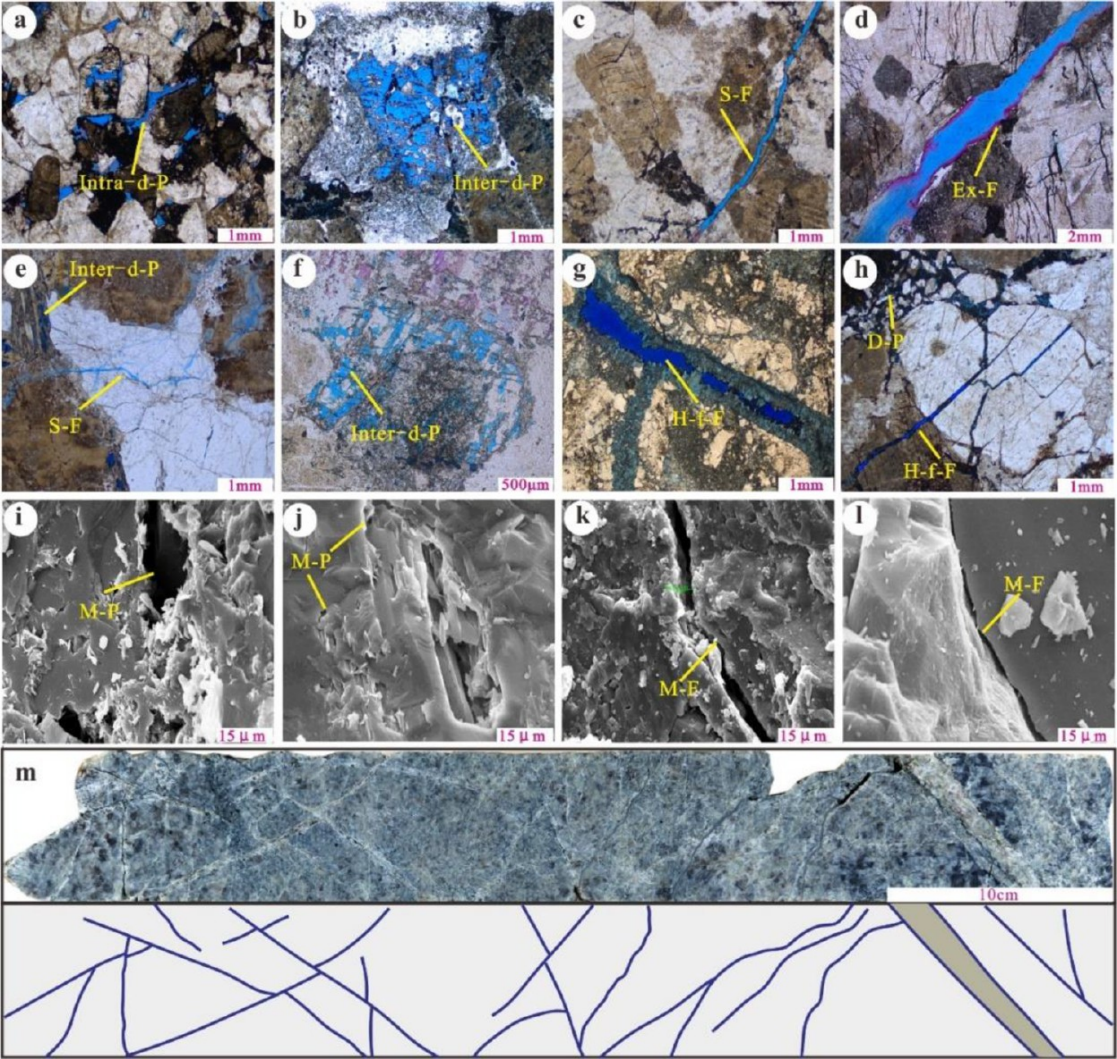
Reservoir space for different types of reservoirs. (a) Type I fracture-pore reservoir, intergranular dissolution pore, well BZ19-6-D, 4429m. (b) Type I fracture-pore reservoir, feldspar intragranular dissolved pore, well BZ19-6-B, 3988.5m. (c) Type I fractured reservoir, structural fracture, well BZ19-6-14,4515m. (d) Type I fractured reservoir, dissolution enlarged fracture, well BZ19-6-G, 4599.23m. (e) Type II fractured reservoir, structural fracture, dissolution pore in mica grain, well JZ20-2-C, 2102.72m. (f) Type II fracture-pore reservoir, feldspar intragranular dissolved pore, well BZ19-6-G, 4815m. (g) Type II fractured reservoir, half-filled fracture, well BZ19-6-H, 4640m. (h) Type II fractured reservoir, half-filled fracture and weak dissolution along the fracture, well BZ19-6-O, 4497m. (i) Type III fracture-pore reservoir, albite intragranular dissolution micropores developed, well BZ19-6-D, 4657m. (j) Type III fracture-pore reservoir, developed dissolution micropores in plagioclase grains, well BZ19-6-D, 4619m. (k) Type III fractured reservoir, microfractures in calcite, well BZ13-2-F, 5128.5 m. (l) Type III fractured reservoir, quartz and feldspar intergranular microfractures, well BZ13-2-E, 4409 m. (m) Core observation macroscopic fracture characteristics, well BZ19-6-L, 5521.8m.

**Figure 6:**
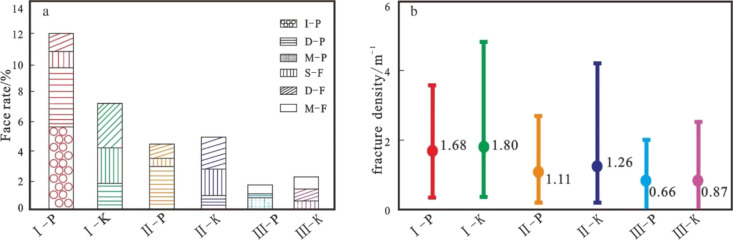
Different types of reservoir space face pore/fracture rates and fracture development rates. (a) Face/slit rates of different types of reservoir space. (b) Electrical imaging logging explains the fracture development rates in different types of reservoirs (P represents a fracture-pore reservoir, and K represents a fracture reservoir).

The type I fracture-pore reservoirs are dominated by intergranular dissolution pores and strongly corroded intragranular dissolution pores. The intergranular dissolution pores are formed by the dissolution of the fine matrix between the weathered gravel and fragmented particles. For example, it can be observed in fractured porous reservoirs such as well BZ19-6-D, that the intergranular dissolution pores formed by the dissolution of the fillers between the feldspar and the quartz particles, as well as the development of the dissolution pores within the feldspar particles, (Figure 5a). Observations of the samples from the BZ19-6-B well show a large area of dissolution along the cleavage cracks in the feldspar particles that formed corrosion pores (Figure 5b), and the observation and statistics of the porosity of the cast thin sections show that the intergranular porosity of the type I fracture-pore type reservoirs ranges from 1.81 to 13.86%, with an average value of 5.52% (Figure 6a). The intragranular porosity rate ranges from 0.78 to 18.24%, with an average of 4.02% (Figure 6a).

The type II fracture-pore reservoirs are mainly composed of intergranular dissolution pores. Compared with the type I pore type reservoirs, type II fracture-pore type reservoirs have no intergranular dissolution pores and the degree of dissolution becomes weaker. The dissolution of feldspar in the BZ19-6-G well (Figure 5f) is obviously weaker than that in the BZ19-6-B well (Figure 5b), and calcite and argillaceous filling can be seen along the feldspar cleavage (Figure 5f). Observation and statistical display of the casting thin slice porosity show that the intragranular porosity of type II fracture-pore type reservoirs ranges from 0.63 to 10.46%, with an average of 2.84% (Figure 6a).

The type III fracture-pore type reservoirs are dominated by dissolution micropores, and there are few pores visible on the thin section scale. Scanning electron microscopy observations show that micropores are mainly dissolution micropores in albite and plagioclase particles (Figure 5i,j). According to the observation statistics of casting thin sections and scanning electron microscopy, the average values of the dissolution pore and the microporous pore rates in type III fracture-pore type reservoirs are 0.77 and 0.66%, respectively (Figure 6a).

Type I fractured reservoirs are mainly characterized by large-scale structural fractures and dissolution expansion fractures. The structural fracture surface is straight and has a large extension and opening (Figure 5c,m) often cutting through mineral particles. The structural fracture surface has characteristics of multiple stages, groups, angles, and sizes (Figure 5m). Observation of core and thin sections shows that its length is between 3 and 22 cm and its opening is between 0.1 and 5 mm. After the formation of some cracks, corrosion occurs along the crack surface to form a dissolution enlarged crack (Figure 5d). The crack surface is irregular, and the dissolution effect increases the surface fracture rate. The observation and statistics of the surface fracture rate of the cast thin sections show that the structural fracture rate of type I fractured reservoirs ranges from 0.69 to 5.66%, with an average value of 2.43% (Figure 6a). The dissolution fracture surface fracture rate ranges from 0.76 to 7.09% with an average value of 2.96% (Figure 6a). This type of reservoir has a relatively high fracture development rate, with an imaging logging fracture density of 0.36–4.83 m^–1^ and an average value of 1.8 m^–1^ (Figure 6b).

Type II fractured reservoirs are mainly characterized by small-scale structural fractures and semifilled fractures. Compared with type I fractured reservoirs, the extension of structural fractures becomes shorter, the opening decreases, and the degree of filling becomes larger. For example, the fracture opening of the JZ20-2-C well is significantly lower than that of well BZ19-6-O (Figure 5e), the fracture length observed by the core and thin section does not exceed 14 cm, and the opening does not exceed 0.3 mm. The semifilled fracture is formed by incomplete dissolution of the previously filled fracture (Figure 5g) or incomplete filling of the structural fracture (Figure 5h). The observation and statistics of the surface fracture rate of the cast thin sections show that the structural fracture rate of type II fractured reservoirs ranges from 0.35 to 4.82%, with an average value of 1.78% (Figure 6a). The semifilled joint surface fracture rate ranges from 0.24 to 4.6% with an average value of 2.15% (Figure 6a). The development rate of fractures in this type of reservoir is moderate, with an imaging logging fracture density of 0.19–4.18 m^–1^ and an average value of 1.26 m^–1^ (Figure 6b).

The type III fractured reservoirs are mainly composed of microcracks within and between the grains (Figure 5k,l). The observation and statistics of the surface fracture rate of the cast thin section by scanning electron microscopy show that the structural fracture rate of type III fractured reservoirs ranges from 0.15 to 1.04% with an average value of 0.56%. The dissolution fracture rate ranges from 0.25 to 1.34%, with an average value of 0.79%. The microfracture rate ranges from 0.19 to 1.40%, with an average value of 0.78% (Figure 6a), and the imaging logging fracture density is 0–2.54 m^–1^, with an average value of 0.87 m^–1^ (Figure 6b).

### 5.2 Pore Structure Characteristics of Different Types of Reservoirs

As an unconventional oil and gas reservoir, metamorphic rock reservoirs have a more complex pore network system, strong heterogeneity, and low porosity and permeability, and the pore structure has a significant impact on the storage and migration of oil and gas. The pore structure refers to the size, shape, and connectivity of the pore throats in metamorphic rock reservoirs. The pore volume, pore size distribution, and other parameters of metamorphic rock reservoirs can be determined by high-pressure mercury injection and nitrogen adsorption experiments to evaluate their pore structure.^22−24^ The experimental samples were divided into two groups, groups A and B, to more intuitively display the differences in the pore structures of different types of reservoirs. Group A is a representative sample of fractured porous reservoirs, and Group B is a representative sample of fractured reservoirs.

#### 5.2.1 High-Pressure Mercury Injection and Pore Size Distribution

**Figure 7:**
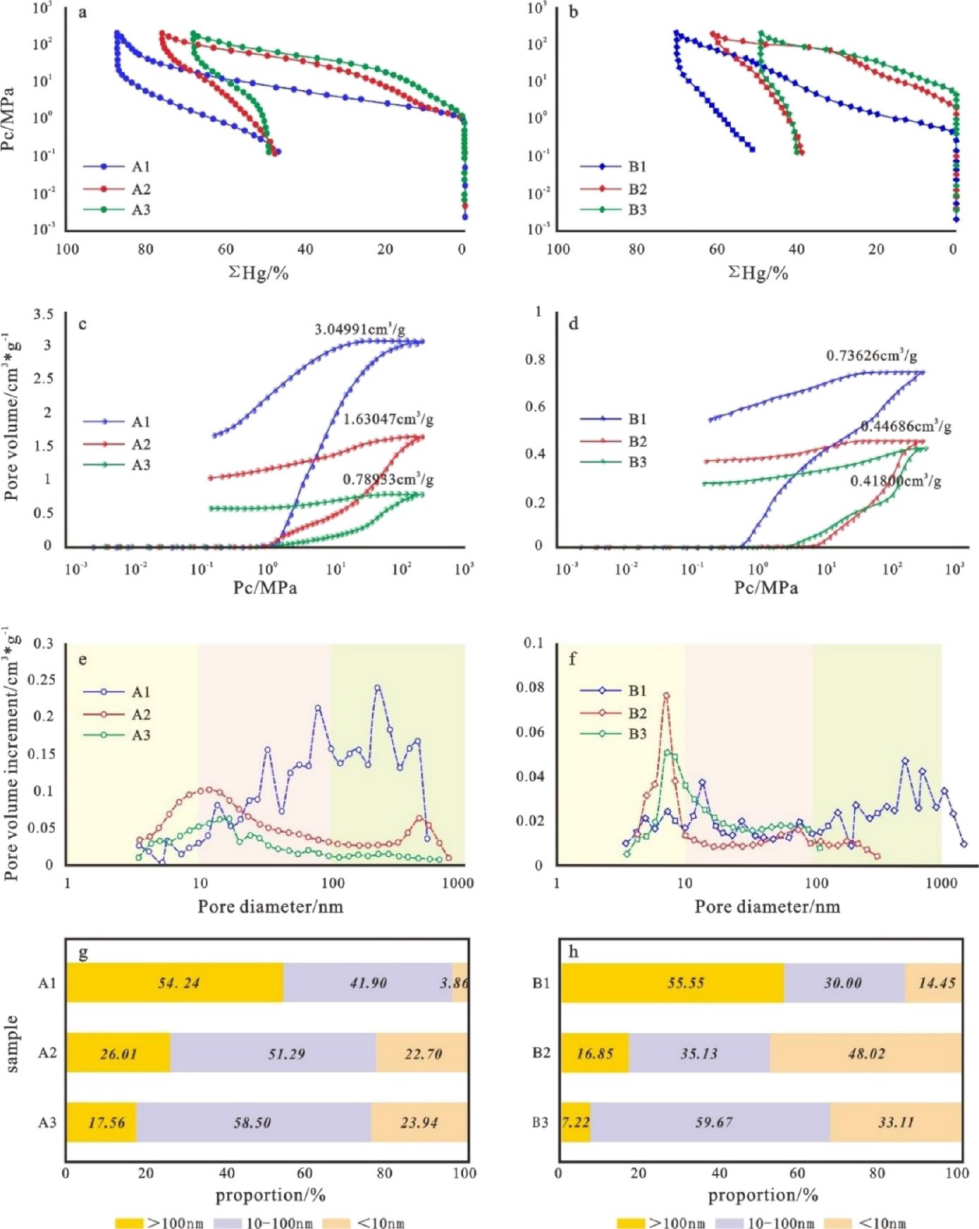
Typical MICP curve and pore size distribution characteristics of six types of reservoirs in the Archean metamorphic buried hills in the Bohai Sea. (a) Characteristics of high-pressure mercury injection curves for representative samples of fracture-pore type reservoirs. (b) Characteristics of high-pressure mercury injection curves for representative samples of fractured reservoirs. (c) Characteristics of pore volume and hysteresis loop in high-pressure mercury injection experiments for representative samples of fracture-pore type reservoirs. (d) Characteristics of pore volume and hysteresis loop in high-pressure mercury injection experiments for representative samples of fractured reservoirs. (e) Pore size distribution characteristics of high-pressure mercury injection experiments for representative samples of fracture-pore type reservoirs. (f) Pore size distribution characteristics of high-pressure mercury injection experiments for representative samples of fractured reservoirs. (g) Different pore size ratios in high-pressure mercury injection experiments for representative samples of fractured porous reservoirs. (h) Different pore size ratios in high-pressure mercury injection experiments for representative samples of fractured reservoirs.

The morphology and characteristic parameters of the mercury injection capillary pressure (MICP) curve can reflect the distribution of the connected pores in rocks.^25,26^Figure 7a,b shows the MICP curve characteristics of six reservoir samples in the study area. The MICP curve of the group A sample exhibits a “platform-like” feature, with a slow curve rise, a relatively small curve slope, and high maximum mercury injection saturation. The MICP of the group B samples exhibits a “fast climbing” characteristic, with a rapid rise in the curve and a decrease in the maximum mercury injection saturation. The mercury removal efficiencies are all below 50%. Figure 7c,d shows that the total MICP curves of the six reservoir samples in the study area all have a wide hysteresis loop. These phenomena reveal that the reservoirs in the study area are developed with open pores, strong heterogeneity, and poor connectivity. At the same time, it is revealed that the fracture-pore type reservoir has a wider oil-and-gas storage space.

There is a significant difference in the mercury intrusion curves between different types of samples. With the deterioration of the physical properties and the reservoir type deteriorating, the mercury intrusion curve moves from the bottom left to the top right. This indicates that there are fewer connected pores with large pore to fine throat types. With the deterioration of the physical properties, the mercury entry pressure gradually increases and the maximum inlet mercury saturation gradually decreases, indicating that the difficulty of mercury entering the pore gradually increases. As the physical properties deteriorate, the skewness coefficient gradually moves away from zero, indicating that the pore throat sorting gradually deteriorates (Figure 7a,b). As the physical properties of the type I to type III samples deteriorate, the pore throat radius distribution transitions from a wide and gentle single peak to a narrow and narrow double peak, and the main peak of the pore size shifts toward the small pore size direction (Figure 7e,f). The proportion of pores larger than 100 nm gradually decreases (Figure 7g,h). The distribution of pore throats affects the quality of reservoirs. This corresponds to the characteristics of the reservoir space in the previous article. As the reservoir deteriorates, intergranular pores, large pores with strong dissolution, and large-scale fractures gradually decrease.

It is noted specifically that in the column of the mercury removal efficiency in Table 2, it is easy to find that the mercury removal efficiency of type II fractured reservoir sample B2 is higher than that of type I fractured reservoir B1. This indicates the significance of the fractures for oil and gas reservoirs. In addition to providing storage space and seepage channels during the reservoir formation process, the positive significance of fractures in the development stage should not be underestimated. Sample B3 illustrates the importance of pore sizes between 10 and 100 nm for oil and gas in the study area (Figure 7h).

**Table 2:** Pore Structure Parameters of Typical High-Pressure Mercury Injection Reservoir Samples

type		number of sample	porosity/%	permeability/×10^–3^ μm^2^	median pressure/MPa	displacement pressure/MPa	median radius/μm	skewness	maximum mercury saturation/%	mercury withdrawal efficiency/%
fracture-pore	I	A-1	8.66	0.262	8.47	1.48	0.087	0.17	87.09	45.78
	II	A-2	5.54	0.095	53.66	1.51	0.014	–0.39	75.81	37.00
	III	A-3	3.10	0.076	69.39	1.71	0.01	–0.37	67.91	27.67
fractured	I	B-1	2.73	1.014	32.34	0.51	0.023	0.22	69.98	27.02
	II	B-2	2.01	0.216	158.06	2.03	0.005	–0.42	60.79	35.59
	III	B-3	2.41	0.021		6.84		–0.33	48.93	18.31

#### 5.2.2 Nitrogen Adsorption and Pore Size Distribution

The nitrogen adsorption method is based on the nitrogen adsorption capacity and relative pressure to obtain parameters such as pore volume and pore size, which reflect the size of the pores. The type, openness, and connectivity of the pores are reflected by the shape and area of the hysteresis loops generated by the adsorption and desorption curves. The nitrogen adsorption method is widely used to characterize the pore structure of porous media.^27,28^ Nitrogen adsorption experiments have been applied in the study of pore structure in shale and coal, and the determinants of hysteresis looping have been clearly analyzed.^29,30^ Nitrogen adsorption is less commonly used in the characterization of metamorphic rock reservoirs. Micropores and microfractures are of great significance to metamorphic rock gas reservoirs in the study area. In this study, the experiment aims to characterize the characteristics of micropores and microfractures on the nanoscale.

**Figure 8:**
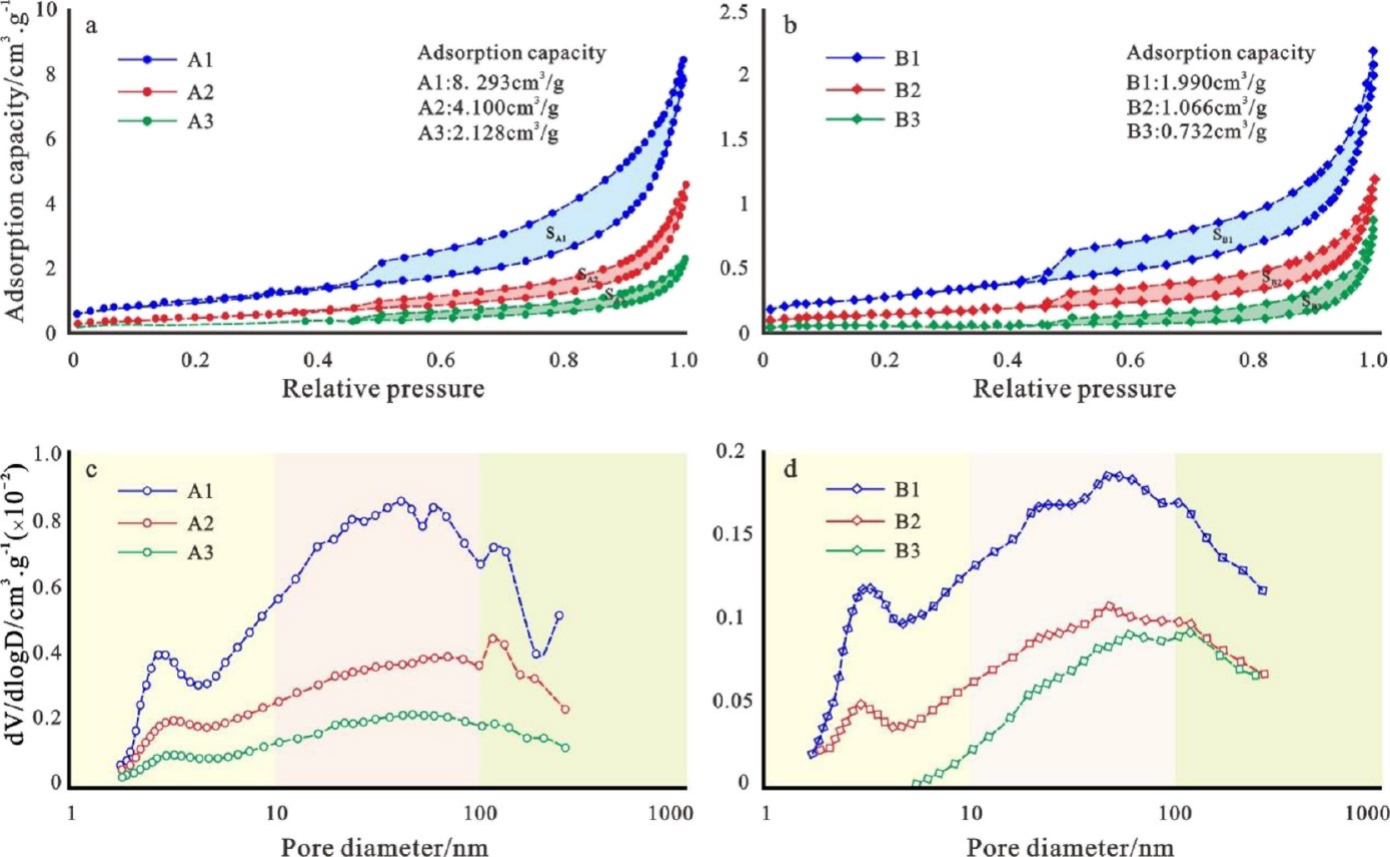
Typical nitrogen adsorption–desorption curves and pore size distribution characteristics of six types of reservoirs in Archean metamorphic buried hills in the Bohai Sea. (a) Characteristics of nitrogen adsorption experiment adsorption and desorption curves for representative samples of fracture-pore type reservoirs. (b) Characteristics of nitrogen adsorption experiment adsorption and desorption curves for representative samples of fractured reservoirs. (c) Pore size distribution of nitrogen adsorption experiments for representative samples of fractured porous reservoirs. (d) Pore size distribution of nitrogen adsorption experiments for representative samples of fractured reservoirs.

Figure 8a,b shows the isothermal adsorption desorption curves of the representative samples from groups A and B. It can be seen that the curves are in a reverse “S″ shape and exhibit characteristics of the three stages of low pressure, transition, and high pressure. ① When *p*/*p*_0_ < 0.05, the isothermal adsorption–desorption curve rises gently and is slightly convex, with single-layer adsorption being the main stage; ② When p/p_0_ is between 0.05 and 0.80, the adsorption capacity slowly increases, and the adsorption changes from adsorption in a single layer to that in multiple layers; ③ When *p*/*p*_0_ > 0.80, the adsorption curve significantly steepens, marking the stage of capillary condensation filling pores. When the relative pressure approaches the saturated vapor pressure, there is no gentle phase, and the adsorption does not reach a saturated state. These characteristics indicate the development of micropores, mesopores, and macropores in the rock samples.

According to the IUPAC classification standard of the isothermal curve, the adsorption isotherms of samples A and B were between the type II and type III reservoirs and the hysteresis loop shows the characteristics of H_3_ and H_4_. H_3_ reflects the flat slit structure, fracture, and wedge structure developed in the storage space of the samples in the study area, and H_4_ reflects the existence of the slit hole of the samples in the study area. Figure 8c,d shows the characterization results of the nitrogen adsorption pore size for three types of fracture-pore types and three types of fracture-type reservoir samples, indicating that the pore size of 10–100 nm is very significant for the oil and gas presence in the study area.

Figure 8a,b shows that the nitrogen adsorption capacity of the group A samples ranges from 2.128 to 8.293 cm^3^/g, while the group B samples have nitrogen adsorption capacities ranging from 0.732 to 1.990 cm^3^/g. Figure 8c,d shows that the corresponding pore volume increment of group A samples under the same pore size is significantly higher than that of group B. This reflects that fracture-pore type reservoirs have a more advantageous pore volume and a stronger oil and gas storage capacity compared to fracture-type reservoirs.

The comparison of the representative sample data from different types of reservoirs in the same group of samples shows the following: ①The isothermal adsorption curves of type I, II, and III reservoir samples gradually reduce the nitrogen adsorption amount under equilibrium pressure; ② S_A1_ > S_A2_ > S_A3_ and S_B1_ > S_B2_ > S_B3_ (S_A1_, S_A2_, S_A3_, S_B1_, S_B2_ and S_B3,_ respectively, represent the hysteresis loop areas of six types of samples, all of which are obtained by integrating the desorption curve based on the adsorption curve); ③The pore volume corresponding to the same pore size gradually decreases (Figure 8c,d). In other words, the type of reservoir changes from good to bad, and the nanoscale pore structure gradually deteriorates as the physical properties deteriorate.

### 5.3 Reservoir Development Mode

In the process of forming buried hill reservoirs through a long geological history, the original rock is affected by geological factors such as tectonic uplift, compression deformation, weathering leaching, erosion, and fracture.^7−13^ Although different scholars have slightly different results in the division of the vertical structure of the differing buried hill reservoirs, they are all divided into two units, namely, weathering crust and buried hill interior, based on the reservoir genesis and are further subdivided into different zones according to the intensity of the weathering and the density of the internal fractures.^10,11,31^ Drawing on previous achievements and analyzing data such as rock cores, thin sections, and logging in the study area, the reservoir is longitudinally divided into a weathered glutenite zone, a weathered leaching zone, a weathered disaggregation zone, an internal fracture zone, and a dense fracture zone. It is particularly noted that the study area extensively develops fractured segments formed by faulting, which can develop in both weathered crusts and buried hill interiors.^10,32^ The types of reservoirs developed in different structural units are different. Taking the BZ 19-6 and BZ 13-2 structural areas as examples, this study analyzed the vertical developments of various reservoirs and established a reservoir distribution pattern of “upper differential, middle continuous, and bottom local aggregation.″

**Figure 9:**
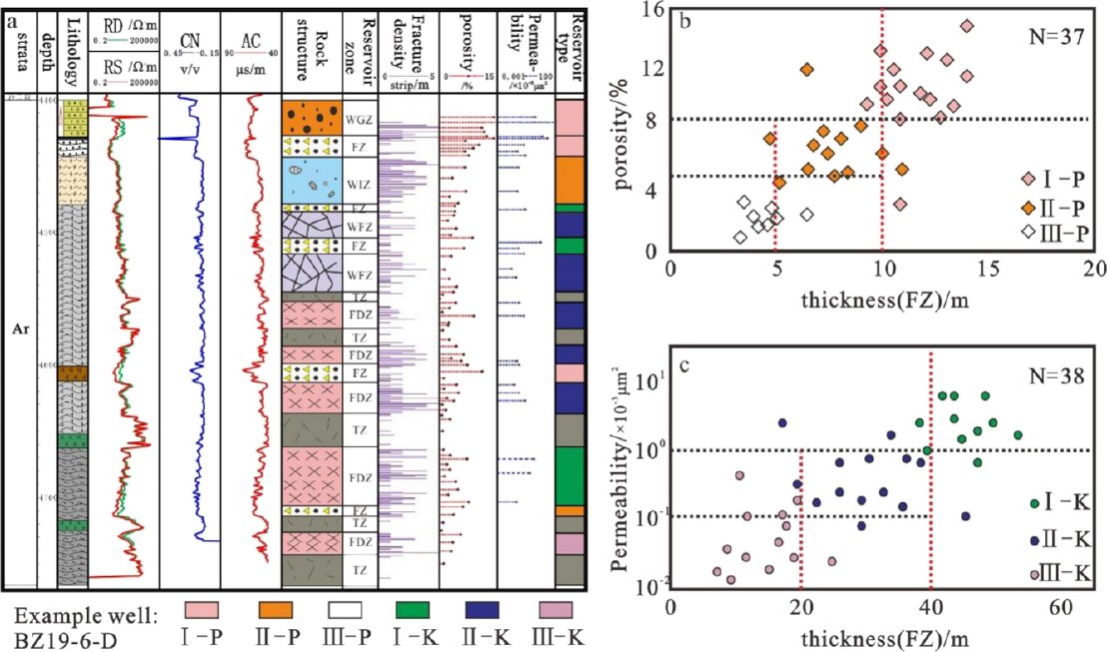
Vertical developments of a single-well reservoir (P represents a fracture-pore reservoir, K represents a fracture reservoir, WGZ represents the weathered glutenite zone, FIZ represents the weathered leaching zone, WFZ represents the weathered disintegration zone, FDZ represents the internal fracture zone, FZ represents the cataclastic section, and TZ represents the dense fracture zone).

Type I fracture-pore reservoirs mainly develop in three parts: weathered glutenite zone, weathered leaching zone of superimposed fractured section, and internal fractured section (>10 m) (Figure 9a,b). The weathered glutenite zone is the product of strong weathering and leaching, and it is a high-quality reservoir space with strong mineral dissolution, more large-scale pore size development, and a high reservoir porosity. The cataclastic section is the reflection of the tectonic movement on bedrock. The thick cataclastic section is produced by deep faults, where atmospheric water migrates along the fault and dissolves, which strengthens the dissolution degree of the weathered leaching zone. The deep hydrothermal fluid migrates along the fault to dissolve the inner cataclastic section. The weathered leaching zone and the inner cataclastic section (>10 m) of the superimposed cataclastic section develop large-scale pore sizes, and it has good reservoir energy capacity and high-quality reservoirs.

Type II fracture-pore reservoirs mainly develop in the weathering leaching zones and the internal cataclastic sections (5–10 m) (Figure 9a,b). With increasing distance from the top surface of the weathered shell, the leaching effect weakens. Small-scale dissolution pores are generated in the weathered leaching zone as oil-and-gas storage spaces. The fractured section of the thin layer is generated by small-scale faults with limited ability to transport fluids and weak dissolution, forming some small-pore-size oil-and-gas storage spaces.

Type III fracture-pore reservoirs mainly develop in the internal fractured section with extremely weak dissolution (<5 m) (Figure 9b), and this type of reservoir mainly uses some dissolution micropores as the storage space, with poor storage capacity and mostly low oil-and-gas production.

Type I fractured reservoirs mainly develop in the weathered and disintegrated zones, with superimposed fragmented segments and thick dense fracture zones (>40 m) (Figure 9a,c). The weathering and disintegration zone is the product of the joint action of tectonic stress and weathering. The development of reticular weathering fractures and superimposed fragmentation sections enhances the dissolution degree and expansion of the atmospheric freshwater on the cracks, which is conducive to oil and gas accumulation and migration. Thick and densely fractured zones are produced by large-scale faults, which have characteristics of high density, large scale, good fracture connectivity within the zone, and high-quality reservoir development.

Type II fractured reservoirs mainly develop in the weathered disintegration zone and the middle dense fracture zone (20–40 m) (Figure 9a,c). The weathered disintegration zone is dominated by undissolved weathering cracks, and the middle dense fracture zone is generated by small fracture activities. The fracture opening and extension length decrease and the storage capacity decreases.

The type III fractured reservoir mainly develops in the thin layer dense fracture zone (<20 m) (Figure 9a,c). The thin layer dense fracture zone is affected by the long-distance fracture activity stress. The fractures have the characteristics of low density, small scale, and local development, and the physical properties of the reservoir are poor, with mostly low yield performance.

**Figure 10:**
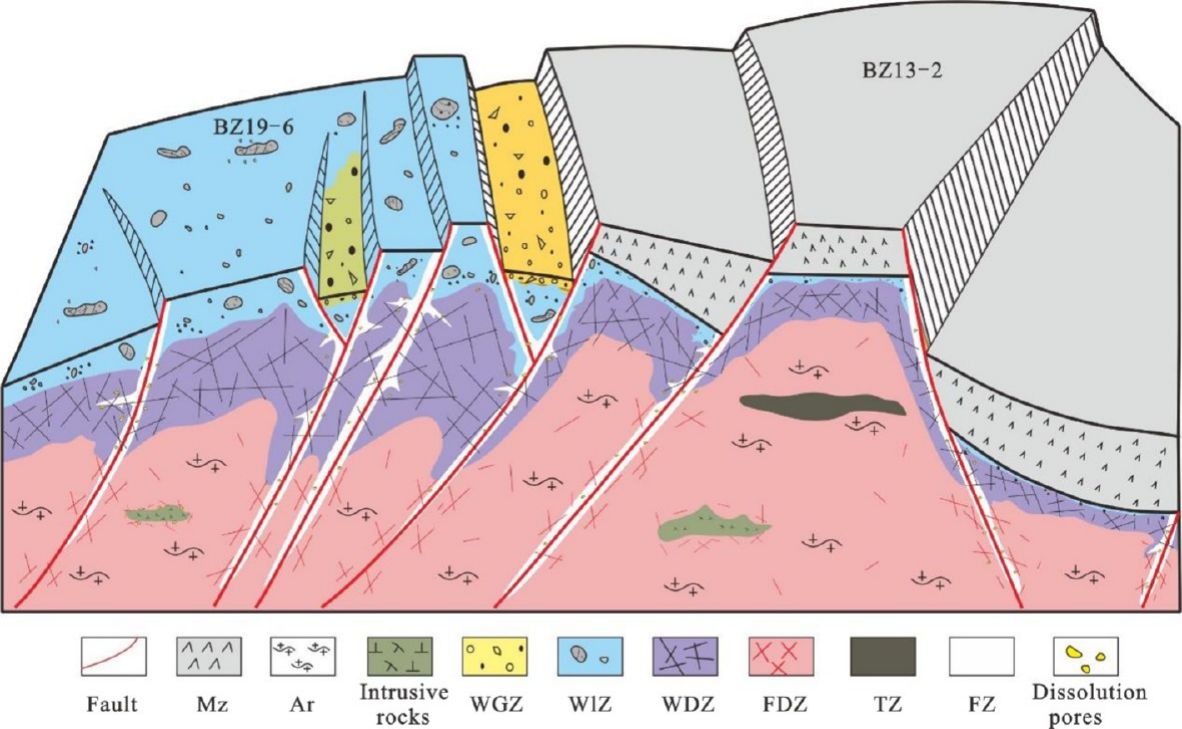
Development model of Archean metamorphic buried hill reservoirs in the Bohai Sea.

The exposure time and paleogeomorphology of buried hills are important factors controlling the sandy conglomerate and leaching belt at the top of the weathered crust. The long exposure time has a strong leaching effect, and the weathered sandy conglomerate is preserved in the lower part of the paleogeomorphology (Figure 10). The weathering effect is weakened when the Mesozoic or Paleozoic strata are overlying and the preexisting top weathered leaching belt reservoir is filled and destroyed (Figure 10). The weathering and disintegration zone in the middle is controlled by tectonic movement and weathering. It is widely and continuously distributed in the study area, and the difference in tectonic activity intensity results in different thicknesses. The reservoir inside the buried hill is controlled by fault activity; the cataclastic section and the dense fracture zone are dendritic under the influence of the fault, and the dense fracture zone around the intrusive body can also form the reservoir (Figure 10).

## 6 Conclusions

The Archean metamorphic buried hill reservoirs in the Bohai Sea can be divided into six types: type I fracture-pore type (Φ > 8%), type I fracture type (*K* > 1 × 10^–3^ μm^2^), type II fracture-pore type (5% < Φ < 8%), type II fracture type (0.1 × 10^–3^ μm^2^ < *K* < 1 × 10^–3^ μm^2^), type III fracture-pore type (2% < Φ < 5%), and type III fracture type (0.01 × 10^–3^ μm^2^ < *K* < 0.1 × 10^–3^ μm^2^). Type I and type II reservoirs are the main sources of industrial flow reservoirs.

The reservoir space of the fracture-pore reservoir is dominated by intergranular and dissolution pores, and the dissolution degree of the reservoir gradually decreases from a type I to type III reservoir. The reservoir space of the fractured reservoirs is dominated by structural fractures and corrosion expansion fractures. The development degree of reservoirs from type I to type III fractures gradually decreases and the dissolution is weakened.

Fracture-pore reservoirs have a larger volume space and stronger oil-and-gas storage capacity than fracture-type reservoirs. The pore diameter of the fracture-pore reservoir ranges from type I to type III and moves from a single peak to a double peak, the peak corresponding pore throat radius moves from large to small, and the proportion of large pore-size pores gradually decreases.

The weathered glutenite zone, the weathered erosion zone of the superimposed cataclastic section, the weathered disintegration zone of the superimposed cataclastic section, and the thick dense fracture zone are the main formation parts of the type I reservoir. The weathering eluviation zone, the weathered disintegration zone, and the middle dense fracture zone are the main forming parts of the type II reservoir. The thin layer dense fracture zone mainly forms a type III reservoir. The spatial distribution of the reservoirs is characterized by 'upper differential, middle continuous, and bottom local aggregation.' The paleogeomorphology, weathering, and tectonic activity are the three major factors that affect the reservoir development model.
